# North African Populations Carry the Signature of Admixture with Neandertals

**DOI:** 10.1371/journal.pone.0047765

**Published:** 2012-10-17

**Authors:** Federico Sánchez-Quinto, Laura R. Botigué, Sergi Civit, Conxita Arenas, María C. Ávila-Arcos, Carlos D. Bustamante, David Comas, Carles Lalueza-Fox

**Affiliations:** 1 Institut de Biologia Evolutiva (CSIC-Universitat Pompeu Fabra), Barcelona, Spain; 2 Department of Genetics, Stanford University, Stanford, California, United States of America; 3 Departament d'Estadística, Universitat de Barcelona, Barcelona, Spain; 4 Centre for GeoGenetics, Natural History Museum of Denmark, University of Copenhagen, Copenhagen, Denmark; University of Florence, Italy

## Abstract

One of the main findings derived from the analysis of the Neandertal genome was the evidence for admixture between Neandertals and non-African modern humans. An alternative scenario is that the ancestral population of non-Africans was closer to Neandertals than to Africans because of ancient population substructure. Thus, the study of North African populations is crucial for testing both hypotheses. We analyzed a total of 780,000 SNPs in 125 individuals representing seven different North African locations and searched for their ancestral/derived state in comparison to different human populations and Neandertals. We found that North African populations have a significant excess of derived alleles shared with Neandertals, when compared to sub-Saharan Africans. This excess is similar to that found in non-African humans, a fact that can be interpreted as a sign of Neandertal admixture. Furthermore, the Neandertal's genetic signal is higher in populations with a local, pre-Neolithic North African ancestry. Therefore, the detected ancient admixture is not due to recent Near Eastern or European migrations. Sub-Saharan populations are the only ones not affected by the admixture event with Neandertals.

## Introduction

Probably the most striking finding derived from the Neandertal genome project [1] was the evidence for admixture between Neandertals and a population of modern humans that left Africa between 80 Kya and 50 Kya subsequently expanding into the rest of the world. The study involved the sequencing and comparison of the Neandertal genome to five modern human genomes: two African (Yoruba and San) and three non-Africans (French, Chinese and Melanesian); all the non-African human genomes shared with Neandertals between 1–4% of their genome, in regions of low recombination placed along ten chromosomes [1]. Additional genomic region introgressions from Neandertals, Denisovans and also putative archaic African hominins have been recently described in Eurasian, Oceanic and even African populations, respectively [2]–[7].

However, an alternative scenario in which the ancestral population of today non-Africans was more closely related to Neandertals than the ancestral population of current Africans due to ancient substructure within the African continent, cannot be totally excluded with the present data [8], although it seems unlikely [9]. In light of this, it is unfortunate that North African individuals have not been included in these admixture analyses, since both the putative African substructure and the admixture are likely to differentially affect North African and sub-Saharan African populations.

The importance of North Africa in the emergence of modern Homo sapiens has been traditionally neglected. However, recent archaeological and paleontological evidence increasingly points to this area as a potential source of out-of-Africa migrations [10],[11]. Recent dating of the characteristic North African lithic industry, called Aterian, has provided much older dates than previously assumed, now ranging from 145 Kya to 40 Kya [12],[13]. These Aterian people made personal ornaments with shells, a sign of modern human symbolic behavior [14]. Morphometric analyses of the 80 Kya Dar es-Soltan skull (Morocco) and of Aterian hominin teeth show similarities with early modern humans from Qafzeh and Skhul (Israel) and with the later skull of Pestera cu Oase (Romania) [15],[16].

Recent genetic analysis of North African populations [17] have found that, despite the complex admixture genetic background, there is an autochthonous genomic component which is likely derived from “back-to-Africa” gene flow older than 12,000 years ago (ya) (i.e., prior to the Neolithic migrations). This local population substratum seems to represent a genetic discontinuity with the earliest modern human settlers of North Africa (those with the Aterian industry) given the estimated ancestry is younger than 40,000 years ago [17]. The estimated time of Neandertal admixture with modern human populations is between 37,000–86,000 years ago [18].

**Table 1:** Average ancestry proportions in North African populations estimated by ADMIXTURE for *k* = 4 different ancestries (the best *k* value determined by cross-validation error).

Population	N	Maghreb*	Europe*	Near East*	Sub-Saharan Africa*
Morocco North	18	0.44	0.31	0.14	0.11
Morocco South	16	0.44	0.13	0.10	0.33
Saharawi	18	0.55	0.17	0.10	0.18
Algeria	19	0.39	0.27	0.16	0.18
Tunisia	18	0.93	0.04	0.02	0.01
Libya	17	0.31	0.28	0.25	0.16
Egypt	19	0.19	0.37	0.30	0.14

* Ancestries are labeled according to the region where the component is the commonest.

The aim of this work was to investigate if this autochthonous North African ancestry bares any traces of the introgression with Neandertals, by applying the f4 ancestry ratio statistic test, previously used to detect Denisovan admixture in Asia [3]. We show that North African populations, like all non-African humans [1], also carry the signature of admixture with Neandertals, and that the real geographical limit for Neandertal admixture is between sub-Saharan groups and the rest.

**Figure 1:**
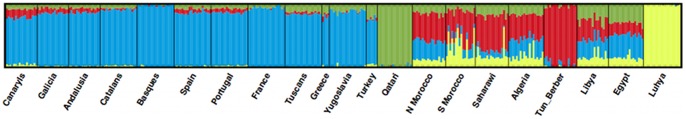
Results of the ADMIXTURE analysis (*k* = 4) with North African populations. ADMIXTURE was performed on a set of European, North African, Near Eastern and Sub-Saharan populations in order to account for the different admixture proportions in North Africa. Tunisians and Saharawi are the North African populations with highest proportion of autochthonous component, whereas the rest of the populations have greater amounts of admixture with neighboring populations.

## Materials and Methods

To ascertain whether or not current North African populations show any signs of Neandertal admixture, we analyzed recently published data of 125 North African individuals genotyped with the Affymetrix 6.0 chip and accounting for 780,000 SNPs were analyzed [17]. Individuals are representative of seven different North African locations (Table 1) spanning from west to east. To have a broader coverage of Eurasia and to allow comparison with Sub-Saharan populations, African and Eurasian populations were included in the analysis [17],[19],[20].

**Figure 2:**
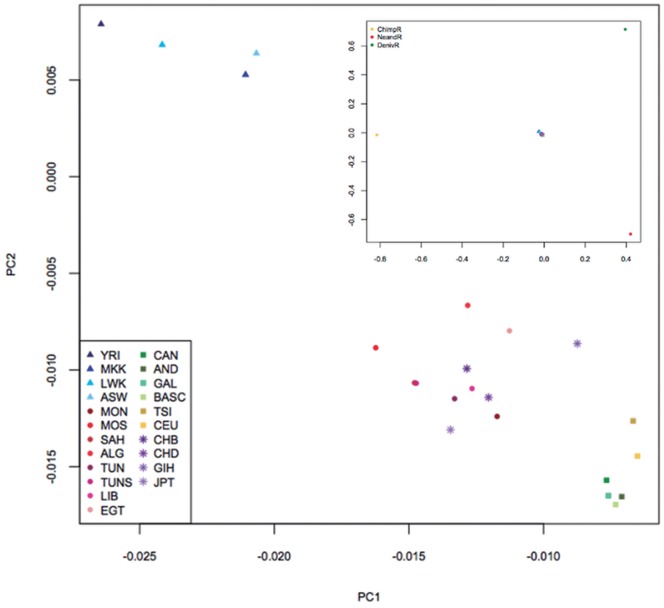
PCA analysis of North African, Sub-Saharan, European and Asian populations. Upper right box: PCA analysis with the African populations (dark blue, Sub-Saharan, light blue, North African), along with three outgroups: chimpanzee, Neandertal and Denisovan. It can be seen that North African populations are placed in the direction of the Neandertal. In the population analysis, the North African groups tend to be placed in an intermediate position between Sub-Saharan and non-African human populations. Population abbreviations are the same as in Table 2.

**Table 2:** Estimates of Neandertal ancestry in North African populations, along with European, Asian and Sub-Saharan African groups.

Population	Code	N	Estimated ancestry	Standard error	Z score
Algeria	ALG	19	44.57%	6.24%	7.15
Tunisia	TUN	17	100.16%	7.18%	13.95
Tunisia 100%	N-TUN	9	138.13%	10.32%	13.39
Egypt	EGT	19	58.45%	5.73%	10.2
Libya	LIB	17	56.36%	5.91%	9.53
Morroco North	MON	18	69.17%	5.37%	12.87
Morocco South	MOS	16	17.90%	6.76%	2.65
Saharawi	SAH	18	50.90%	6.30%	8.08
Canary Island*	CAN	17	101.44%	4.62%	21.95
China Beijing	CHB	84	193.43%	16.41%	11.79
China	CHD	85	195.41%	16.62%	11.76
Japan	JPT	86	201.18%	17.22%	11.69
Texas Indu Gupti	GIH	88	84.37%	5.77%	14.62
Andalusia*	AND	15	118.66%	5.34%	22.22
CEU	CEU	112	100.00%	0.00%	inf
Tuscan	TSI	88	94.90%	3.12%	30.4
Basque	BASC	20	129.48%	6.34%	20.44
Galicia*	GAL	16	115.86%	4.82%	24.06
Yoruba	YRI	113	0.00%	0.00%	nan
Luyha	LWK	90	−14.89%	4.50%	−3.31

European HapMap CEU was selected as the known to have experience Neandertal admixture, Chimpanzee as the out-group and Sub-Saharan YRI as the non Neandertal admixed population. * unpublished data.

**Figure 3:**
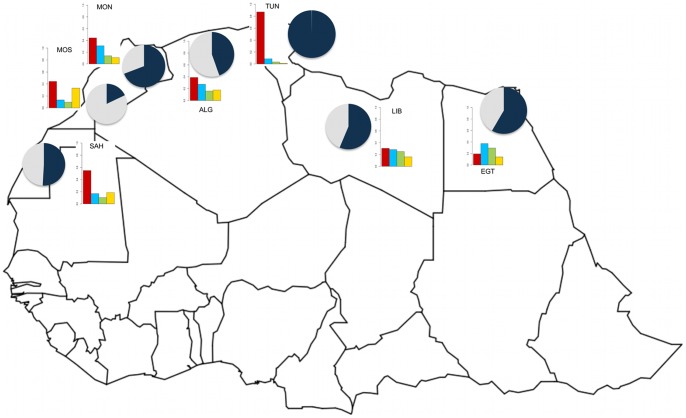
Neandertal genetic introgression in North African populations as a fraction of that found in Europeans. Relative proportion of Neandertal ancestry for each population is presented as the dark blue section of the pies on a map of North Africa. Additionally, each population is also represented as a barplot of the different geographic genetic components; in red: North African, in blue: Sub-Saharan, in green: European, in yellow: Near East. Populations are shown as having Neandertal ancestry if the estimates are more than two standard errors from zero. Full name descriptions of these population labels are found in Table 2.

**Table 3:** Pearson's product – moment correlation between modern human ancestry in North African groups and Neandertal admixture.

Comparison		Correlation (p-value)
European, Near Eastern vs. Neandertal	With Tunisia	−0.44 (0.8384)
	Without Tunisia	0.49 (0.1572)
European, Near Eastern, North Africa vs. Neandertal	With Tunisia	0.91 (0.0020*)
	Without Tunisia	0.90 (0.0068*)

In order to compare the human SNP data to the Neandertal, bam read files from all Neandertal samples from the UCSC ftp site (ftp://hgdownload.cse.ucsc.edu/gbdb/hg18/neandertal/seqAlis) were downloaded and merged. Base and mapping quality filters reported in previous studies were implemented in the analysis [2],[21]. To avoid any confusion with ancient DNA postmortem modifications, C-T and G-A human – ancient hominin nucleotide sites, were discarded. For all sequencing data, a single read was randomly sampled for each individual at positions overlapping the array SNPs coordinates. Furthermore all human and Neandertal data were merged with sequence data from chimpanzee (CGSC 2.1/Pantro), and data were further processed to control for strand misidentification [3], to conform a final data set of 142,720 SNPs.

North African populations have a complex genetic background. In addition to an autochthonous genetic component, they exhibit signals of European, sub-Saharan and Near Eastern admixture as previously described [17]. Moreover, the use of genotype data can suffer from potential biases that arise from discovering SNPs in a limited number of individuals, thus resulting in enrichment of common alleles, particularly in the populations from which the discovery panel was constructed [22],[23] (in the present case would be a bias towards European populations). Two challenges arise from these effects: first, patterns of gene flow detected between Neandertal and North Africans could be the consequence of subsequent admixture between North Africans and other modern human populations and second, the ascertainment bias towards European and East Asian populations could magnify differences in signals of Neandertal gene flow in individuals with high Sub-Saharan ancestry compared to individuals with high European ancestry.

In order to overcome these problems we initially assessed the different genetic components in North African populations using an unsupervised clustering algorithm, ADMIXTURE [24], on a sample set of around 50,000 SNPs that included all North African individuals, together with populations of European, Near Eastern and Sub-Saharan origin [17],[25],[26].

As a first approach to establish the relationship between North African populations and Neandertal, a projected Principal Component Analysis (PCA) was performed. In addition to the chimpanzee and the Neandertal genomes, data from the Denisova genome were downloaded and merged in this case resulting in 111,991 SNPs (after filtering for strand bias SNPs and ancient DNA miscoding lesions). Given that the ancient hominin and chimpanzee genomes have been originally sequenced at low coverage no SNP polymorphism data are available, and therefore individuals were considered at the haplotype level only. First, a PCA was generated using Neandertal, chimpanzee, and Denisova. Then, SNP loadings for the first two components were used to project the sample set of modern humans.

Next, we aimed at estimating the amount of Neandertal admixture in North African populations using the f4 ancestry ratio test [27]. Although a previous simulation study [28] suggested that the analysis of SNP data from arrays can provide biased results in admixture estimates, there is more recent evidence supporting that f4 ancestry ratio statistic is unaffected by those biases [3]. The f4 ancestry ratio test measures the proportion of archaic hominin genetic fraction in a modern human population as a fraction of the known amount of archaic introgression in another modern human population. Consequently, the f4 ancestry ratio test basically measures the correlation in allele frequency differences between two populations used as outgroups (e.g., chimpanzee and Neandertal), a Sub-Saharan African population (Yorubans) and the X-tested population, normalized by the correlation in allele frequency differences between chimpanzee, Neandertal, a Sub-Saharan African group (Yorubans) and a human population previously known to have experienced Neandertal admixture (in this case, CEU) [1]. If Yorubans and X descend from a single ancestral population without any subsequent admixture with Neandertals, then the allele frequency differences between Yorubans and X must have arisen solely after their separation from their common ancestor; the two frequency differences should be uncorrelated and thus the f4 ancestry ratio statistic should have an expected value of zero.

Finally, a block jackknife [29],[30] approach was used to estimate standard errors; blocks were separated by dropping each non-overlapping five cM stretch of the genome in turn, and studying the variance of each statistic of interest to obtain a approximately normal distributed standard error [25]. Further combinations (e.g. San instead of Yoruban and Chinese instead of CEU) were also calculated to test the consistency of the results (Table S1).

## Results and Discussion

We ran ADMIXTURE for *k* equal 2 to 7 and obtained CV errors, and determined that the best *k* (the one with lowest cross-validation error) is *k* = 4. Results (Figure 1) are coincident with those previously published [17] and show that North Morocco, Libya and Egypt carry high proportions of European and Near Eastern ancestral components, whereas Tunisian Berbers and Saharawi are those populations with highest autochthonous North African component. Particularly, ten Tunisian individuals have more than 99% of their genome assigned to North African ancestry and therefore have been analyzed separately (subsequently referred to as N-TUN) from the overall Tunisian population.

In the PCA analysis (Figure 2) Eurasian populations are the closest to Neandertals among modern humans, which is in agreement with previous studies [1]. Sub-Saharan Africans are, as expected, more distant to Neandertal, whereas North African individuals are placed between these two groups. North African individuals with the highest Sub-Saharan African component (as detected by ADMIXTURE) are distant from Neandertal and closer to Sub-Saharan populations. It is interesting to notice that the North African populations closer to Neandertals are populations with a large known European or Near Eastern admixture, but also the Tunisians that have an almost complete autochthonous North African genetic component.

The results of the f4 ancestry ratio test (Table 2 and Table S1) show that North African populations vary in the percentage of Neandertal inferred admixture, primarily depending on the amount of European or Near Eastern ancestry they present (Table 1). Populations like North Morocco and Egypt, with the highest European and Near Eastern component (∼40%), have also the highest amount of Neandertal ancestry (∼60–70%) (Figure 3). On the contrary, South Morocco that exhibits the highest Sub-Saharan component (∼60%), shows the lowest Neandertal signal (20%). Interestingly, the analysis of the Tunisian and N-TUN populations shows a higher Neandertal ancestry component than any other North African population and at least the same (or even higher) as other Eurasian populations (100–138%) (Figure 3).

Some results of the estimated ancestry in Table 2 are higher than 100%. Because the amount of Neandertal admixture provided by this statistic is in relation to the fraction found in another population, populations with more than 100% values, have more than the observed Neandertal admixture levels found in the “source population” used for comparison (i.e CEU). On the other hand, a negative f4 ancestry ratio value such as that one observed for the Luyha in Table 2 could have several explanations. One possibility is that it reflects an artifact of ascertainment bias on SNP arrays. Ascertainment bias is likely to affect the joint information from Europeans and East Asians, since SNP arrays are most commonly designed based on information from these populations. On the other hand it could also reflect a more complex demographic history (i.e population structure between the populations being compared) than previously assumed.

Subsequently, we aimed to compare the results revealed by ADMIXTURE and by the f4 ancestry ratio statistic in an attempt to corroborate that the signal of Neandertal admixture revealed in North African populations is not caused by Eurasian admixture. For this purpose, we performed a Pearson correlation test between the ancestry proportions estimated with ADMIXTURE and the proportions of Neandertal admixture estimated by the f4 ancestry ratio test. Specifically, we tested the correlation between a) both European and Near Eastern components and Neandertal admixture and b) European, Near Eastern and North African admixture components and Neandertal admixture. If signals of gene flow from Neandertals were due exclusively to the European and the Near Eastern components, we would expect that the correlation should significantly decrease in test b), when the North African component is included. On the contrary, the Pearson correlation test reaches significance only when the North African component is included, which is maintained even when Tunisians are removed from the analysis (Table 3).

Overall, the correlation analysis and the f4 ancestry ratio statistic show that the North African component actually contributes to the signal of gene flow from Neandertals. Given that the North African autochthonous ancestry seems to be 12,000–40,000 years old [17], our hypothesis is that this ancestral population was descendant from the populations that first interbreed with Neandertals about ∼37,000–86,000 years ago [18] somewhere in the Middle East. Nonetheless further analyses in populations around the contact areas are needed to confirm this hypothesis.

A previous study [26] observed that the similarity to Neandertals increases with distance from Africa and suggested this could be explained by SNP ascertainment bias plus a strong genetic drift in East Asian populations. Nonetheless more complex, population-biased, ascertainment schemes might have additional effects (i.e bottlenecks), but these are not expected to significantly increase the rate of false positives in admixture tests [31]. The Tunisian population has been reported to be a genetic isolate [17] so it is plausible that part of the signal detected is actually due to genetic drift. However, this should not affect the other North African groups in our study. Finally, given that SNP arrays are based on common alleles and probably the relevant admixture information is encoded within the rare and very rare alleles, the potential bias, if anything, will underestimate ancient hominid admixture signals, as shown in previous studies [2],[3].

With the current data, however, it is not possible to discard the ancient African substructure hypothesis [8]. Although ours and some previous results [9] tend to favor the admixture hypothesis as the most plausible one, we think that a complete clarification of this issue can only be achieved with a Neandertal high coverage genome, such as this recently achieved for Denisova [32]. This, and sequencing data of North African populations, especially those with a high autochthonous component, may help elucidate more precisely the interbreeding process with Neandertals. In any case, our results show that Neandertal genomic traces do not mark a division between African and non-Africans but rather a division between Sub-Saharan Africans and the rest of modern human groups, including those from North Africa.

## Supporting Information

Table S1 **Stability of the Neandertal admixture estimates.** We present each population's estimate ancestry, the standard error in the estimate, and the Z score for different combinations of Sub-Saharan and non-African populations. O (Out-group), BP (Benchmark population, i.e. population which didn't experience any introgression from Neandertals) and SP (Source population i.e. populations in which the amount of introgression from Neandertal is known).
(DOC)

## References

[1] Green RE, Krause J, Briggs AW, Maricic T, Stenzel U, et al (2010) A draft sequence of the Neandertal genome. Science 328: 710–722. 20448178 10.1126/science.1188021 PMC5100745

[2] Reich D, Green RE, Kircher M, Krause J, Patterson N, et al (2010) Genetic history of an archaic hominin group from Denisova Cave in Siberia. Nature 468: 1053–1060. 21179161 10.1038/nature09710 PMC4306417

[3] Reich D, Patterson N, Kircher M, Delfin F, Nandineni MR, et al (2011) Denisova Admixture and the First Modern Human Dispersals into Southeast Asia and Oceania. Am J Hum Genet 89: 516–528. 21944045 10.1016/j.ajhg.2011.09.005 PMC3188841

[4] Abi-Rached L, Jobin MJ, Kulkarni S, McWhinnie A, Dalva K, et al (2011) The Shaping of Modern Human Immune Systems by Multiregional Admixture with Archaic Humans. Science 334: 89–94. 21868630 10.1126/science.1209202 PMC3677943

[5] Hammer MF, Woerner AE, Mendez FL, Watkins JC, Wall JD (2011) Genetic evidence for archaic admixture in Africa. Proc Natl Acad Sci USA 108: 15123–15128. 21896735 10.1073/pnas.1109300108 PMC3174671

[6] Rasmussen M, Guo X, Wang Y, Lohmueller KE, Rasmussen S, et al (2011) An Aboriginal Australian Genome Reveals Separate Human Dispersals into Asia. Science 334: 94–98. 21940856 10.1126/science.1211177 PMC3991479

[7] Yotova V, Lefebvre JF, Moreau C, Gbeha E, Hovhannesyan K, et al (2011) An X-linked haplotype of Neandertal origin is present among all non-African populations. Mol Biol Evol 28: 1957–1962. 21266489 10.1093/molbev/msr024

[8] Eriksson A, Manica A (2012) Effect of ancient population structure on the degree of polymorphism shared between modern human populations and ancient hominins. Proc Natl Acad Sci USA 109: 13956–13960. 22893688 10.1073/pnas.1200567109 PMC3435202

[9] Yang MA, Malaspinas AS, Durand EY, Slatkin M (2012) Ancient structure in Africa unlikely to explain Neanderthal and non-African genetic similarity. Mol Biol Evol Apr 18. [Epub ahead of print]. 10.1093/molbev/mss117 PMC3457770 22513287

[10] Osborne AH, Vance D, Rohling E, Barton N, Rogerson M, et al (2008) A humid corridor across the Sahara for the migration “Out of Africa” of early modern humans 120,000 years ago. Proc Natl Acad Sci USA 105: 16444–16447. 18936490 10.1073/pnas.0804472105 PMC2575439

[11] Balter M (2011) Was North African the launch pad for Modern Human migrations? Science 331: 20–23. 21212332 10.1126/science.331.6013.20

[12] Barton RNE, Bouzouggar A, Collcutt SN, Schwenninger J-L, Clark-Balzan L (2009) OSL dating of the Aterian levels at Grotte de Dar es-Soltan I (Rabat, Morocco) and possible implications for the dispersal of modern Homo sapiens. Quaternary Sci Rev 28: 1914–1931.

[13] Garcea EAA (2010) The spread of Aterian peoples in North Africa. In: Garcea, E.A.A., editor. South-Eastern Mediterranean Peoples Between 130,000 and 10,000 years ago. Oxford: Oxbow Books.

[14] Bouzouggar A, Barton RNE, Vanhaeren M, D'Errico F, Collcutt S, et al (2007) 82,000-year-old shell beads from North Africa and implications for the origins of modern human behavior. Proc Natl Acad Sci USA 104: 9964–9969. 17548808 10.1073/pnas.0703877104 PMC1891266

[15] Harvati K, Hublin J-J (2012) Morphological continuity of the face in the late Middle and Upper Pleistocene hominins from northwestern Africa – A 3D geometric morphometric analysis. In: Hublin J-J, McPherron S, editors. Modern Origins: A North African Perspective. Dordrecht: Springer.

[16] Hublin J-J, Verna C, Bailey S, Smith T, Olejniczak A, et al.. (2012) Dental evidence from the Aterian human populations of morocco. In: Hublin J-J, McPherron S, editors. Modern Origins: A North African Perspective. Dordrecht: Springer.

[17] Henn BM, Botigué LR, Gravel S, Wang W, Brisbin A, et al (2012) Genomic ancestry of North Africans supports back-to-Africa migrations. Plos Genetics 8: e1002397. 22253600 10.1371/journal.pgen.1002397 PMC3257290

[18] Sankararaman S, Patterson N, Li H, Pääbo S, Reich D (2012) The date of interbreeding between Neandertals and modern humans. arXiv: 1208.2238v1. 10.1371/journal.pgen.1002947 PMC3464203 23055938

[19] International HapMap 3 Consortium, Altshuler DM, Gibbs RA, Peltonen L, Altshuler DM, et al (2010) Integrating common and ra genetic variation indiverse human populations. Nature 467: 52–57. 20811451 10.1038/nature09298 PMC3173859

[20] Novembre J, Johnson T, Bryc K, Kutalik Z, Boyko AR, et al (2008) Genes mirror geography within Europe. Nature 456: 98–101. 18758442 10.1038/nature07331 PMC2735096

[21] Skoglund P, Malmström H, Raghavan M, Stora J, Hall P, et al (2012) Origins and Genetic Legacy of Neolithic Farmers and Hunter-Gatherers in Europe. Science 336: 466–469. 22539720 10.1126/science.1216304

[22] Kuhner MK, Beerli P, Yamato J, Felsenstein J (2000) Usefulness of single nucleotide polymorphism data for estimating population parameters. Genetics 156: 439–447. 10978306 10.1093/genetics/156.1.439 PMC1461258

[23] Albrechtsen A, Nielsen FC, Nielsen R (2010) Ascertainment biases in SNP chips affect measures of population divergence. Mol Biol Evol 27: 2534–2547. 20558595 10.1093/molbev/msq148 PMC3107607

[24] Alexander DH, Novembre J, Lange K (2009) Fast model-based estimation of ancestry in unrelated individuals. Genome Res 19: 1655–1664. 19648217 10.1101/gr.094052.109 PMC2752134

[25] Hunter-Zinck H, Musharoff S, Salit J, Al-Ali KA, Chouchane L, et al (2010) Population genetic structure of the people of Qatar. Am J Hum Genet 87: 17–25. 20579625 10.1016/j.ajhg.2010.05.018 PMC2896773

[26] Lao O, Lu TT, Nothnagel M, Junge O, Freitag-Wolf S, et al (2008) Correlation between genetic and geographic structure in Europe. Curr Biol 18: 1241–1248. 18691889 10.1016/j.cub.2008.07.049

[27] Reich D, Thangaraj K, Patterson N, Price AL, Singh L (2009) Reconstructing Indian population history. Nature 461: 489–494. 19779445 10.1038/nature08365 PMC2842210

[28] Skoglund P, Jakobsson M (2011) Archaic human ancestry in East Asia. Proc Natl Acad Sci USA 108: 18301–18306. 22042846 10.1073/pnas.1108181108 PMC3215044

[29] Kunsch HK (1989) The jackknife and the bootstrap for general stationary observations. Ann Stat 17: 1217–1241.

[30] Busing F, Meijer E, Van Der Leeden R (1999) Delete-m jackknife for unequal m. Stat Comput 9: 3–8.

[31] Durand EY, Patterson N, Reich D, Slatkin M (2011) Testing for ancient admixture between closely related populations. Mol Biol Evol 28: 2239–2252. 21325092 10.1093/molbev/msr048 PMC3144383

[32] Meyer M, Kircher M, Gansauge MT, Li H, Racimo F, et al.. (2012) A High-Coverage Genome Sequence from an Archaic Denisovan Individual. Science. Aug 31. [Epub ahead of print]. 10.1126/science.1224344 PMC3617501 22936568

