# Supplementary material for: North African Populations Carry the Signature of Admixture with Neandertals
Source: PLoS One. 2012 Oct 17;7(10):e47765. doi: 10.1371/journal.pone.0047765 (PMC3474783; doi:10.1371/journal.pone.0047765)
Supplement: Table S1 — Stability of the Neandertal admixture estimates. We present each population's estimate ancestry, the standard error in the estimate, and the Z score for different combinations of Sub-Saharan and non-African populations. O (Out-group), BP (Benchmark population, i.e. population which didn't experience any introgression from Neandertals) and SP (Source population i.e. populations in which the amount of introgression from Neandertal is known). (DOC) [file pone.0047765.s001.doc]

|  |  | **O** | Chimpanzee | | **O** | Chimpanzee | | **O** | Chimpanzee | |
| --- | --- | --- | --- | --- | --- | --- | --- | --- | --- | --- |
|  |  | **BP** | San | | **BP** | LWK | | **BP** | YRI | |
|  |  | **SP** | CEU | | **SP** | CEU | | **SP** | CHB | |
| Code | N | Ratio | Std. Error | Z-score | Ratio | Std. Error | Z-score | Ratio | Std. Error | Z-score |
| ALG | 19 | 26% | 10% | 2.58 | 52% | 5% | 10.38 | 23% | 4% | 5.35 |
| TUN | 17 | 100% | 10% | 10.4 | 100% | 6% | 16.05 | 51% | 5% | 10.85 |
| N-TUN | 9 | 151% | 15% | 9.96 | 133% | 9% | 15.5 | 71% | 5% | 13.73 |
| EGT | 19 | 44% | 9% | 4.96 | 64% | 5% | 13.55 | 30% | 4% | 6.9 |
| LIB | 17 | 42% | 9% | 4.47 | 62% | 5% | 12.52 | 29% | 4% | 6.76 |
| MON | 18 | 59% | 8% | 7.61 | 73% | 5% | 15.96 | 36% | 4% | 8.08 |
| MOS | 16 | -10% | 13% | -0.78 | 29% | 5% | 5.45 | 9% | 4% | 2.37 |
| SAH | 18 | 34% | 10% | 3.5 | 57% | 5% | 11.14 | 26% | 4% | 6.06 |
| CAN | 17 | 102% | 6% | 16.44 | 101% | 4% | 25.21 | 52% | 5% | 11.35 |
| CHB | 84 | 225% | 25% | 8.9 | 181% | 13% | 13.97 | 100% | 0% | Inf |
| CHD | 85 | 228% | 26% | 8.88 | 183% | 13% | 13.94 | 101% | 1% | 79.6 |
| JPT | 86 | 236% | 27% | 8.81 | 188% | 13% | 13.94 | 104% | 2% | 57.69 |
| GIH | 88 | 79% | 8% | 10.13 | 86% | 5% | 17.23 | 44% | 4% | 10.46 |
| AND | 15 | 125% | 8% | 16.5 | 116% | 5% | 25.61 | 61% | 5% | 13.21 |
| CEU | 112 | 100% | 0% | inf | 100% | 0% | inf | 52% | 4% | 11.8 |
| TSI | 88 | 93% | 4% | 22.04 | 96% | 3% | 35.37 | 49% | 4% | 11.17 |
| BASC | 20 | 140% | 9% | 14.78 | 126% | 5% | 23.89 | 67% | 5% | 14.14 |
| GAL | 16 | 121% | 7% | 17.77 | 114% | 4% | 27.68 | 60% | 5% | 13.15 |
| YRI | 113 | -34% | 13% | -2.65 | 13% | 3% | 3.8 | 0% | 0% | Nan |
| LWK | 90 | -54% | 15% | -3.71 | 0% | 0% | nan | -8% | 2% | -3.71 |
